# Skeletal Muscle Function Deficit Longitudinal Associations With Inflammation and Caloric Intake in Elderly. The InCHIANTI Study

**DOI:** 10.1002/jcsm.70353

**Published:** 2026-08-05

**Authors:** Matteo Candeloro, Chiara Candita, Raffaello Pellegrino, Giada Mariano, Ettore Porreca, Giovanni Barassi, Roberto Paganelli, Angelo Di Iorio, Toshiko Tanaka, Luigi Ferrucci

**Affiliations:** ^1^ Department of Innovative Technologies in Medicine & Dentistry University “G. d'Annunzio” Chieti‐Pescara Italy; ^2^ Center for Disability, Rehabilitation, and Sports Medicine (CARES), University “G. d'Annunzio” Chieti‐Pescara Italy; ^3^ Università LUM “Giuseppe Degennaro” Casamassima Bari Italy; ^4^ Saint Camillus International Medical University Rome Italy; ^5^ Longitudinal Studies Section, Translational Gerontology Branch National Institute on Aging, National Institutes of Health, USA Baltimore Maryland USA

**Keywords:** aging, caloric intake, interleukin‐6, longitudinal study, muscle function

## Abstract

**Background:**

The Skeletal Muscle Function Deficit (SMFD) score integrates muscle mass, strength, power and quality into a single construct that evaluates the multidimensional status of muscle health. The aim of the study is to investigate the longitudinal associations of systemic inflammation and caloric intake with SMFD‐score trajectories in older adults from the InCHIANTI study.

**Methods:**

Data were obtained from the InCHIANTI population‐based longitudinal cohort. A total of 1035 community‐dwelling older adults (mean age 74.97 ± 7.39 years; 44.25% men) contributed 3196 repeated observations across four study waves. SMFD‐score (range 0–20) was derived from sex‐specific quintiles of lower‐limb muscle mass, muscle density, grip strength and lower‐limb power; participants unable to perform a test received a score of 0 for that component. Interleukin‐6 (IL‐6) and caloric intake (kcal/day) were assessed at all study waves. Longitudinal associations between time‐varying IL‐6 tertiles, caloric intake tertiles and SMFD‐score trajectories were evaluated using linear mixed‐effects models adjusted for baseline age and sex and time‐varying multimorbidity and body mass index.

**Results:**

Across follow‐up, SMFD‐score declined significantly from 9.60 ± 4.20 at baseline to 7.07 ± 4.00 at the last wave (*p* < 0.001), whereas IL‐6 increased from 3.65 ± 2.50 pg/mL to 4.39 ± 3.10 pg/mL (*p* < 0.001). Mean caloric intake showed a non‐significant upward trend (1912.83 ± 562.37 to 1976.00 ± 580.54 kcal/day; *p* = 0.07). In mixed‐effects models, higher IL‐6 levels were associated with accelerated SMFD decline over time (IL‐6 tertile × time interaction β = −0.31 ± 0.02; *p* < 0.001). Higher caloric intake was associated with higher SMFD‐score and a slower functional decline (caloric intake tertile main effect β = 1.19 ± 0.16; *p* < 0.001; caloric intake tertile × time interaction β = 0.24 ± 0.03; *p* < 0.001). In the model including the combined IL‐6 × caloric intake × time interaction was statistically significant (β = 0.14 ± 0.04; *p* < 0.001), indicating that higher caloric intake partially attenuated the negative longitudinal association between IL‐6 and SMFD‐score.

**Conclusions:**

SMFD‐score captures longitudinal muscle function decline during aging and reflects the opposing influences of inflammation and caloric intake. These findings support the SMFD‐score as a clinically relevant construct for identifying potentially modifiable determinants of muscle health and functional decline in older adults.

## 1 Introduction

Aging represents one of the pivotal drivers for the gradual decline of the skeletal muscle and the complexity of muscle changes exceeds the straightforward loss of muscle mass alone [1]. The concept of the Skeletal Muscle Function Deficit (SMFD) has been introduced to capture the multidimensional alterations of muscle during aging, including variation in mass, strength, power and quality in one integrative construct [2, 3]. The SMFD‐score, as proposed recently by Di Iorio et al. [4] allows for a more comprehensive estimation of skeletal muscle biological aging by integrating structural, metabolic and functional domains [3, 5, 6, 7].

Muscle mass, density, strength and power decline with age and the decline in their trajectories inversely correlates with age [8]. However, these complex changes are not fully explained by the reduction in muscle mass alone and suggest other factors including metabolic derangement, nutritional intake and inflammation are involved as well [9, 10].

Caloric intake may represent an important modulator of muscle function; for example, inadequate caloric intake in older adults may blunt anabolic responses, contributing to muscle mass and function decline [11]; indeed, whereas moderate caloric restriction appears to improve muscle quality, extreme caloric restriction has been linked to an accelerated progression of muscle failure especially among frail persons [11, 12, 13] and in cancer diagnosed patients [14].

Inflammaging, a chronic low‐grade inflammatory condition, has been identified as one of the key biological correlates of the aging muscle [15]. Elevated pro‐inflammatory molecules (IL‐6, TNF‐α), as well as higher serum C‐reactive protein (CRP) levels, are consistently linked to lower muscle performance as well as lower muscle density [16]. Moreover, in the InCHIANTI study, our group previously reported that immunological resilience defined as the ability to preserve or rapidly restore immune function is associated with muscle power, mass, density and strength [6, 17, 18, 19].

Inflammatory status and caloric intake influence one another: suboptimal caloric intake may exacerbate systemic inflammation, whereas adequate caloric and macronutrient supply can attenuate pro‐inflammatory responses, even in frail older adults [20]. This interplay between aging, nutrition and inflammation may contribute to a self‐perpetuating cycle of physiological decline [21].

The aim of this study is to examine the potential association between aging, caloric intake and inflammatory state with SMFD‐score in a population‐based, age‐ and sex‐representative Italian longitudinal cohort from the InCHIANTI study.

## 2 Methods

The InCHIANTI study has been previously described in detail (Ferrucci et al. 2000). Briefly, the study was conceived and implemented by the Laboratory of Clinical Epidemiology at the Italian National Institute of Research and Care of Aging (INRCA, Florence) (Figure S1). Ethical approval was obtained from the Italian National Institute of Research and in the United States the study was granted exemption by the Institutional Review Board of the National Institutes of Health Intramural Research Program (Exemption #11976). Written informed consent was collected from all participants at every study wave. Recruitment occurred between 1998 and 2000, with assessments repeated approximately every 3 years through 2018. The cohort was drawn from two demographically distinct municipalities of Tuscany, one semi‐urban area near Florence and one rural community in the Chianti area.

The variables of interest were measured at baseline and during each of the follow‐up waves.

### 2.1 Muscle Composition Assessment

Peripheral quantitative computed tomography (pQCT) of the tibia scans were obtained using an XCT 2000 scanner (Stratec Medizintechnik, Pforzheim, Germany) following standard procedures [22]. Post‐acquisition image processing was performed with BonAlyse software (BonAlyse Oy, Jyväskylä, Finland) [23]. Cross‐sectional muscle and fat areas were calculated at 38% and 66% of tibial length from the distal end, respectively, providing estimates of calf muscle area (cm^2^), fat area (cm^2^) and muscle density (mg/cm^3^) [22].

### 2.2 Muscle Strength

Isometric grip strength was evaluated using a hydraulic hand dynamometer (Baseline; Smith & Nephew). Isometric handgrip strength was assessed using a manual dynamometer following a standardized protocol. Participants were seated with the tested arm supported on a bench and the elbow flexed at approximately 45°. Before testing, the examiner screened for pain or recent conditions affecting the hands, deferring the test if necessary. The dynamometer handle was individually adjusted to ensure proper hand positioning (proximal interphalangeal joints at ~90°). After a practice trial to ensure familiarity and comfort, participants were instructed to perform maximal effort contractions on command. Participants performed two maximal efforts with each hand and the highest value from the four trials was used in analyses [24].

### 2.3 Lower‐Limb Power Measurement

Peak lower limb extension power was assessed using the Nottingham Power Rig (Medical Engineering Unit, University of Nottingham, UK) [25]. Subjects sat upright with arms crossed, one foot on the floor and the other on the pedal of the device. The knee angle reached approximately 15 flexion at the end of the pedal stroke. After two familiarization trials, participants executed maximal voluntary extensions with each leg until no further increase in peak power was observed. The highest value obtained was recorded as maximal lower‐limb power [8].

### 2.4 Skeletal Muscle Function Deficit (SMFD) and SMFD‐Score

SMFD was operationalized by ranking lower‐limb muscle mass, muscle density, grip strength and power into sex‐specific quintiles derived from the pooled dataset including all available observations across waves (Table S1). Thus, repeated measurements from the same participants at different follow‐up visits contributed to the quintile distribution. For each domain, values ranged from one (lowest quintile) to five (highest quintile). Participants unable to perform a test received a score of zero for that domain. The composite SMFD‐score, representing global muscle function, ranged from 0 to 20 with lower values indicating worse muscle function [4].

### 2.5 Inflammatory State

Complete and differential white blood cell counts were obtained at the Clinical Chemistry and Microbiology Laboratory of SS. Annunziata Hospital (Florence, Italy) using automated haematology analysers. Different analysers were employed at successive waves: Haematology SE 9000 (Sysmex, Kobe, Japan) at baseline; Coulter LH 750 (Beckman Coulter, Brea, California) at first follow‐up; and Sysmex XE 2100 (DASIT, Milan, Italy) thereafter. Serum high‐sensitivity CRP was quantified by immunonephelometry (BN II Nephelometer, Dade Behring Inc., Deerfield, Illinois). Baseline interleukin‐6 (IL‐6) concentrations were determined in duplicate using an ultra‐sensitive enzyme‐linked immunosorbent assay (ELISA) (CytoScreen Human IL‐6; BioSource International Inc., Camarillo, California, USA). Results were calculated based on a regression‐derived calibration curve specific to the assay platform. At subsequent follow‐up examinations, plasma IL‐6 levels were quantified using a high‐sensitivity solid‐phase sandwich ELISA (Quantikine HS Human IL‐6 Immunoassay; R&D Systems, Minneapolis, Minnesota, USA), following the manufacturer's analytical protocol [26].

### 2.6 Multimorbidity Assessment

Chronic diseases were identified using predefined algorithms integrating medical history, examination, laboratory data and medication review. Diagnoses included major cardiovascular, metabolic and respiratory conditions (e.g., myocardial infarction, heart failure, diabetes, chronic obstructive pulmonary disease, stroke, osteoarthritis and cancer). A multimorbidity index was computed as the total number of conditions present at each visit and across follow‐up waves.

### 2.7 Caloric Intake

Caloric intake was evaluated using the Italian‐validated version of the European Prospective Investigation into Cancer and Nutrition (EPIC) food frequency questionnaire [27, 28, 29]. The instrument comprises two sections: one describing general dietary habits and meal frequency and another documenting the consumption frequency and portion size. Because self‐administration proved unreliable in older adults, trained interviewers conducted the assessments. Nutrient and total caloric intake values were automatically computed using EPIC‐specific software. The validity of this method in the InCHIANTI population was confirmed by direct comparison with 7‐day weighed dietary records [9].

### 2.8 Statistical Analysis

Descriptive data are shown as mean ± standard error and as absolute numbers and percentages, for continuous and categorical variables, respectively, at each study wave. Differences in continuous variables across study waves were evaluated using linear mixed models with random intercepts and slopes, whereas differences in categorical variables were assessed using chi‐squared tests.

Longitudinal associations between SMFD‐score and time‐varying tertiles of inflammatory status and caloric intake were analysed using linear mixed‐effects models. These models allowed simultaneous estimation of within‐person change in SMFD‐score over time and between‐person differences in baseline SMFD‐score and in the rate of change over follow‐up. All models were estimated using maximum likelihood (ML) with the PROC MIXED procedure in SAS. Time was modelled as a continuous variable indexed by study wave. Random intercepts were included to account for individual‐specific differences in baseline SMFD‐score and random slopes were tested to capture heterogeneity in longitudinal trajectories over time. Models were fitted sequentially following a pre‐specified, hierarchical model‐building strategy with increasing complexity. First, an unconditional growth model (Model A) was fitted to study the average trajectory of SMFD‐score over time and to quantify between‐subject variability. Second, time‐varying predictors were introduced, including tertiles of IL‐6 and caloric intake, modelled as fixed effects (Model B). Third, interaction terms between each exposure and time were specified to allow the rate of change in SMFD‐score to vary according to IL‐6 and caloric intake (Model C). Fourth, models allowing random slopes for selected exposure‐by‐time effects were fitted to assess inter‐individual variability in the longitudinal impact of inflammation and energy intake on SFMD‐score trajectories (MODEL D). Fifth, two fully adjusted models were fitted separately, one with IL‐6 tertiles and the other with caloric intake tertiles as the primary exposure. Both models included baseline age and sex, as well as time‐varying covariates for multimorbidity, cigarette smoking status, alcohol‐drinking habits, self‐report physical activity level and body mass index (BMI) (Model E). Moreover, to evaluate potential bias introduced by assigning a score of zero to participants unable to perform a test, we conducted a sensitivity analysis in which all analyses were repeated after excluding individuals with SMFD‐score equal to zero. Eventually, to evaluate the combined longitudinal effect of tertiles of distribution of inflammation and caloric intake, an extended model simultaneously included IL‐6 tertiles, caloric intake tertiles, time and their interaction terms. This model specified two‐way interactions between each exposure and time, as well as a three‐way interaction (IL‐6 × caloric intake × time) to formally test whether longitudinal SMFD‐score trajectories differed according to the combined time‐varying inflammatory and nutritional profiles.

Model comparison was performed using the Akaike Information Criterion (AIC), with lower values indicating improved fit. Statistical significance was settled for *p*‐values < 0.05.

## 3 Results

Overall, 1453 adults were enrolled in the original InCHIANTI cohort. For the current analysis, 1035 participants with complete baseline data and up to three follow‐up evaluations were included, generating 3196 person‐observations. Temporal Trends in demographic, inflammatory and nutritional variables are summarized in Table 1. Briefly, mean age at baseline was 74.97 ± 7.39 years and 458 (44.25%) subjects were male.

**TABLE 1:** Descriptive characteristics of the study population across study waves.

	Baseline	Follow‐up 1	Follow‐up 2	Follow‐up 3	*p*
N	1035	835	739	587	
Chronological age (years)	75.48 ± 7.39	77.18 ± 7.01	78.97 ± 6.60	80.41 ± 5.99	< 0.001
Baseline age (years)	74.97 ± 7.39	73.62 ± 7.01	72.36 ± 6.60	70.74 ± 5.98	< 0.001
Male sex *n* (%)	458 (44.25)	365 (43.71)	311 (42.08)	251 (42.76)	0.19
BMI	27.46 ± 4.11	26.47 ± 3.98	27.18 ± 4.14	27.15 ± 4.17	< 0.002
CRP (μg/mL)	5.37 ± 9.58	4.52 ± 9.83	5.30 ± 9.81	4.98 ± 9.86	0.41
IL‐6 (pg/mL)	3.65 ± 2.50	3.57 ± 2.85	4.35 ± 3.16	4.39 ± 3.10	< 0.001
Caloric intake (Kcal/day)	1912.83 ± 562.37	1933.10 ± 565.01	1958.90 ± 564.68	1976.00 ± 580.54	0.07
Multimorbidity (*n*)	2.15 ± 1.53	2.68 ± 1.71	3.03 ± 1.77	3.42 ± 1.72	< 0.001
SMFD‐score	9.60 ± 4.20	9.55 ± 4.73	8.70 ± 4.61	7.07 ± 4.00	< 0.001
Smoking habits					< 0.001
Never	612 (59.1)	484 (58.0)	433 (58.6)	337 (57.4)	
Former smoker	281 (27.2)	259 (31.1)	248 (33.6)	218 (37.1)	
Actually	142 (13.7)	91 (10.9)	58 (7.8)	32 (5.5)	
Alcohol					< 0.001
Teetotaler	160 (15.5)	305 (36.5)	269 (36.4)	209 (35.6)	
Mild (≤ 2 glasses)	555 (53.6)	383 (45.9)	370 (50.1)	280 (47.7)	
Heavy (> 2 glasses)	320 (30.9)	147 (17.6)	100 (13.5)	98 (16.7)	
Physical activity					< 0.001
No physical activity	224 (21.6)	231 (27.7)	318 (43.0)	302 (51.5)	
Light	435 (42.0)	401 (48.0)	285 (38.6)	227 (38.7)	
Moderate/intense	376 (36.3)	203 (24.3)	136 (18.4)	58 (9.9)	

*Note:* Baseline age (of participants present at each wave). Results are displayed as mean ± standard error or number and percentage as appropriate.

Abbreviations: BMI, body mass index; CRP C‐reactive protein; IL‐6, Interleukin‐6; MLR, monocyte to lymphocyte ratio; NLR, neutrophil to lymphocyte ratio; SMFD‐score, skeletal muscle function deficit score.

### 3.1 Temporal Trends

Markers of systemic inflammation exhibited heterogeneous but coherent patterns. CRP levels displayed a wide inter‐individual variability and no temporal change (*p*‐value = 0.41), whereas IL‐6 increased steadily across follow‐ups (*p*‐value < 0.001).

Caloric intake exhibited a mild mean increase, rising from 1912.83 ± 562.37 kcal/day at baseline to 1976.00 ± 581.54 kcal/day at the last follow‐up; however, these mean differences did not reach statistical significance (*p*‐value = 0.07).

Multimorbidity increased, from an average of 2.15 ± 1.53 diseases to 3.42 ± 1.72 (*p*‐value < 0.001), confirming the progressive accumulation of chronic diseases while aging.

Finally, the SMFD‐score showed a relevant downward trajectory, declining from 9.60 ± 4.20 at baseline to 7.07 ± 4.00 at the last follow‐up (*p*‐value < 0.001).

### 3.2 Longitudinal Determinants of SMFD‐Score Trajectories

#### 3.2.1 SMFD‐Score Trajectories

In MODEL A (the unconditional model), the SMFD‐score decreased across follow‐up (γ_10_ = −1.41 ± 0.05; *p*‐value < 0.001) and the variance components indicated substantial heterogeneity both within (δ^2^_e_ = 6.12 ± 0.24; *p*‐value < 0.001) and between individuals as well as in the rate of change over time (δ^2^₁ = 0.24 ± 0.11; *p* = 0.01) (Table 2, Model A).

**TABLE 2:** Linear Mixed‐Effects Models of SMFD‐score trajectories over time according to IL‐6 tertiles.

		Model A	Model B	Model C	Model D	Model E
Fixed effects						
Intercept	γ_00_	11.52 ± 0.15***	12.95 ± 0.22***	11.90 ± 0.39***	10.49 ± 0.20***	1.96 ± 0.82**
Time	γ_10_	−1.41 ± 0.05***	−1.23 ± 0.05***	−0.76 ± 0.15***	—	—
IL‐6 tertiles	γ_20_	—	−0.73 ± 0.08***	−0.26 ± 0.16	0.34 ± 0.10***	0.06 ± 0.09
IL‐6 tertiles × Time	γ_30_	—	—	−0.21 ± 0.06***	−0.52 ± 0.02***	−0.31 ± 0.02***
Variance components						
Within person	δ^2^_e_	6.12 ± 0.24***	5.83 ± 0.24***	5.79 ± 0.24***	6.01 ± 0.26***	6.11 ± 0.24***
Intercept	δ^2^_0_	14.15 ± 1.24***	13.28 ± 1.18***	13.70 ± 1.21***	—	—
In rate of change	δ^2^_1_	0.24 ± 0.11*	0.28 ± 0.11**	0.27 ± 0.11**	—	—
In IL‐6 tertiles	δ^2^_2_	—	—		0.30 ± 0.45	0.06 ± 0.42
In IL‐6 tertiles × Time	δ^2^_3_	—	—	—	0.05 ± 0.02**	0.03 ± 0.01*
AIC		17 273	16 391	16 382	16 372	15 154

*Note:* Model A represents the unconditional growth model. Model B includes IL‐6 tertiles as a fixed effect. Model C additionally includes the interaction between Il‐6 tertiles and time. Model D allows both the intercept and the IL‐6 tertile × time effect to vary randomly, in addition to fixed effects. Model E represents the fully adjusted model, including baseline age and sex, as well as time‐varying covariates for multimorbidity, Body Mass Index (BMI), Cigarette smoking status, Alcohol‐Drink habits, self‐report physical activity level and caloric intake tertiles.

* *p* < 0.05.

** *p* < 0.01.

*** *p* < 0.001.

#### 3.2.2 IL‐6 and SMFD‐Score Trajectories

Table 2 presents the results of linear mixed‐effects models examining SMFD‐score trajectories over time across IL‐6 tertiles.

**FIGURE 1:**
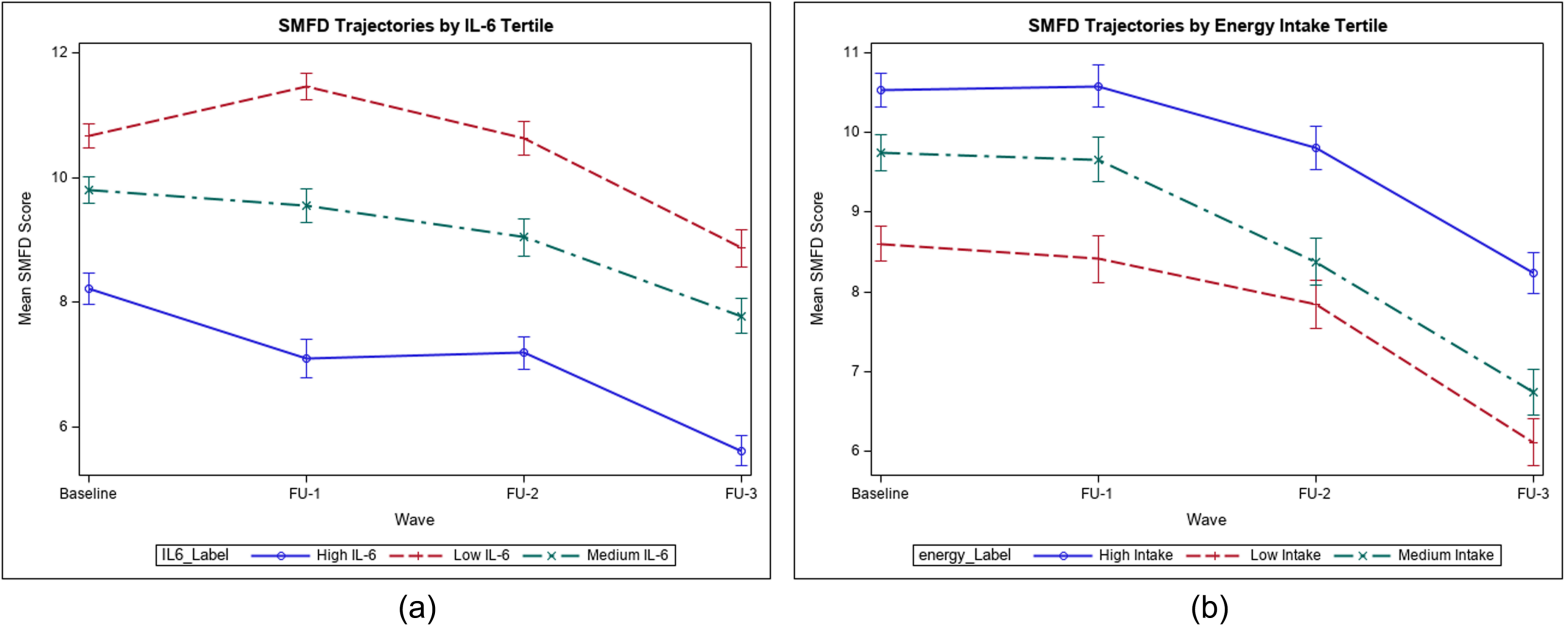
SMFD‐score trajectories by IL‐6 (Panel A) and by caloric intake tertiles (Panel B).

When IL‐6 tertiles was included as a time‐varying predictor (Table 2, Model B), a significant main effect was observed (γ_20_ = −0.73 ± 0.08; *p*‐value < 0.001) as illustrated in Figure 1‐Panel A whereas the rate of decline was similar across IL‐6 tertiles. In MODEL C, the main effect of IL‐6 tertiles (γ_20_ = −0.26 ± 0.16; *p*‐value = 0.10) reflected differences in SMFD‐score at baseline and was not statistically significant. As shown in Figure 1, Panel A, group differences in SMFD‐score emerged primarily over follow‐up rather than at baseline. Consistently, a negative IL‐6 tertile * time interaction was observed (γ_30_ = −0.21 ± 0.06; *p*‐value < 0.001) (Table 2, Model C), indicating an accelerated decline in SMFD‐score at higher IL‐6 levels. In Model D, IL‐6 tertiles were modelled as fixed effects, representing the average population‐level association and with random slopes to account for inter‐individual variability in longitudinal response to inflammation. In this model, the IL‐6 tertiles × time interaction remained statistically significant (γ_30_ = −0.52 ± 0.02; *p*‐value = 0.001), indicating a faster decline in SMFD‐score over time among individuals with higher IL‐6 tertiles, with substantial heterogeneity across subjects (Table 2, Model D). In the fully adjusted model (Table 2, Model E) the negative IL‐6 tertiles × time interaction persisted (γ_30_ = −0.31 ± 0.02; *p*‐value < 0.001), supporting a robust association between higher inflammatory burden and accelerated SMFD‐score decline over time. The progressive reduction in AIC from Model A (17,273) to Model E (15,154) improved model fit with increasing complexity.

We conducted a sensitivity analysis excluding participants with an SMFD‐score of zero to minimize the risk that these observations disproportionately influenced the estimated associations (Table S2); the direction and overall strength of the associations remained consistent with the primary analysis, with only small differences in the magnitude of the estimates.

#### 3.2.3 Caloric Intake and SMFD‐Score Trajectories

Table 3 reports the results of linear mixed‐effects models evaluating the association between energy intake tertiles and SMFD‐score trajectories over time. The unconditional growth model was not repeated, as it was identical to that reported in Table 2 (Model A).

**TABLE 3:** Linear Mixed‐Effects Models of SMFD‐score trajectories over time according to energy intake tertiles.

		Model B	Model C	Model E
Fixed effects				
Intercept	γ_00_	9.11 ± 0.32***	9.62 ± 0.39***	1.67 ± 0.81*
Time	γ_10_	−1.40 ± 0.05***	−1.66 ± 0.12***	—
Caloric intake tertiles	γ_20_	1.28 ± 0.15***	1.02 ± 0.19***	1.19 ± 0.15***
Caloric intake tertiles × Time	γ_30_	—	0.14 ± 0.06*	0.24 ± 0.03***
Variance components				
Within person	δ^2^_e_	6.17 ± 0.24***	6.17 ± 0.25***	6.15 ± 0.26***
Intercept	δ^2^_0_	13.44 ± 1.21***	13.27 ± 1.21***	7.98 ± 0.99***
In rate of change	δ^2^_1_	0.22 ± 0.11*	0.21 ± 0.11*	0.19 ± 0.11**
AIC		17 109	17 070	15 169

*Note:* Model B includes caloric intake tertiles as a fixed effect. Model C additionally includes the interaction between caloric intake tertiles and time. Model E represent the fully adjusted model, including baseline age and sex, as well as time varying covariates for multimorbidity, Body Mass Index (BMI), Cigarette smoking status, Alcohol‐Drink habits, self‐report physical activity level and IL‐6 tertiles.

* *p* < 0.05.

** *p* < 0.01.

*** *p* < 0.001.

In Model B, caloric intake tertiles were included as a fixed effect, showing that higher caloric intake was associated with higher SMFD‐score (γ _20_ = 1.28 ± 0.15, *p* < 0.001). In Model C, the interaction between caloric intake tertiles × time was introduced and remained positive (γ_30_ = 0.14 ± 0.06, *p* = 0.01), indicating a slower decline in SMFD‐score over time among participants with higher caloric intake. Due to model identifiability constraints, random effects were specified for the intercept and time slope only; therefore, models including random slopes for exposure‐related effects (Model D) were not fitted for caloric intake.

In the fully adjusted model (Model E), higher energy intake remained associated with higher SMFD‐score (γ_20_ = 1.19 ± 0.15, *p* < 0.001) and the caloric intake × time interaction remained significant (γ_30_ = 0.24 ± 0.03, *p* < 0.001), independent of age, sex, BMI and multimorbidity, cigarette smoking status, alcohol‐drink habits and self‐report physical activity level. Figure 1 Panel B illustrates SMFD‐score trajectories according to caloric intake tertiles over the study period.

Also in this case, we conducted a sensitivity analysis excluding participants with an SMFD‐score of zero (Table S3) and the direction and overall strength of the associations remained consistent with the primary analysis, with only small differences in the magnitude of the estimates.

#### 3.2.4 IL‐6, Caloric Intake and SMFD‐Score Trajectories

**FIGURE 2:**
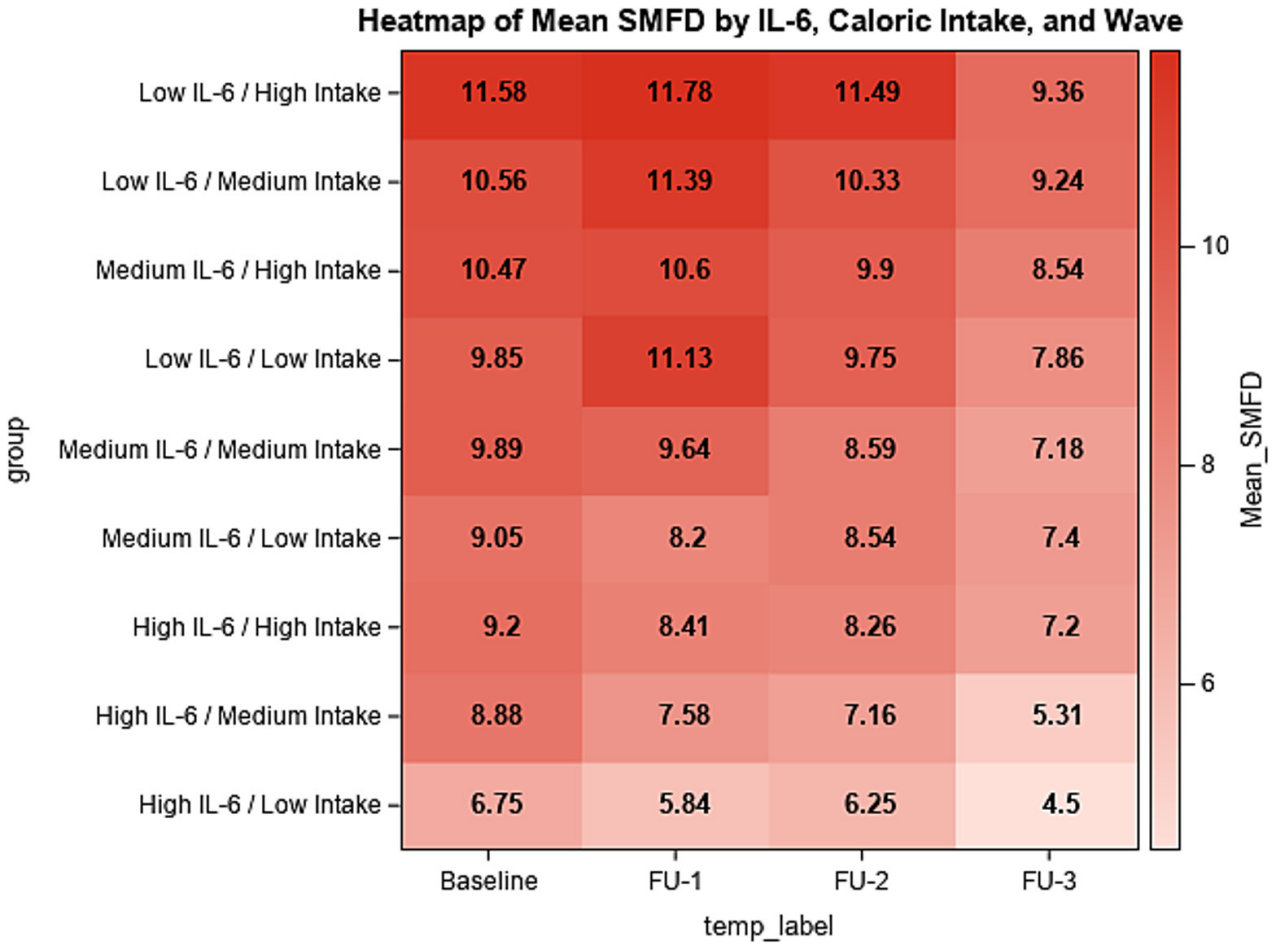
Heatmap of the joint association between tertile of distribution of IL‐6 and caloric intake with SMFD‐score. Footnote Figure 2: Each column represents a time wave. Darker red represents higher mean value of the SMFD‐score; each rectangle is the result of IL‐6 and caloric intake combination. Abbreviation: FU—follow up.

The joint longitudinal association between Il‐6 tertiles, caloric intake tertiles and time across the four study waves is illustrated in Figure 2, with corresponding model estimates reported in Table S4.

Although SMFD‐score declined over time in all groups, the magnitude of decline varied according to the combined inflammatory and nutritional profile. This heterogeneity was supported by a significant IL‐6 × caloric intake × time interaction (0.14 ± 0.04; *p*‐value < 0.001), indicating that SMFD‐score trajectories depend on the combined, time‐varying effects of inflammation and caloric intake.

## 4 Discussion

The main findings of this study show that the decline in SMFD‐score over time is strongly associated with both systemic inflammation and nutritional factors, supporting the view that muscle aging, as captured by the SMFD‐score, is a multifactorial process extending beyond the loss of muscle mass or strength alone. Higher serum levels of IL‐6 were associated with lower SMFD‐scores, whereas greater caloric intake was linked to better muscle function.

Our findings are consistent with previous evidence indicating that inflammaging contributes to the catabolic shift observed in aging skeletal muscle [30, 31]. IL‐6 has a dual biological role, acting not only as a pro‐inflammatory cytokine in chronic conditions but also as a myokine released during muscle contraction; therefore, circulating levels observed in epidemiological settings likely reflect the balance between chronic low‐grade inflammation and physiological responses to muscle activity. Although physical activity may transiently increase IL‐6 levels through exercise‐induced myokine release, in the present study physical activity was included in the fully adjusted models and did not change the strength of the associations between inflammation and SMFD.

Elevated IL‐6 and TNF‐α impair anabolic pathways (i.e., PI3K/Akt/mTOR), while activating NF‐κB and ubiquitin‐proteasome systems, therefore promoting proteolysis and myofibrillar degradation [32]. The SMFD framework integrates multiple muscle domains (strength, power, quality and mass) into a single construct that is capable of capturing the biological complexity of muscle aging [5].

The potential advantage of the SMFD‐score over analyses focused on individual muscle domains lies in its ability to capture the cumulative and multidimensional nature of muscle aging. Rather than representing a single physiological attribute, the SMFD‐score summarizes several interconnected manifestations of age‐related muscle decline into a unified construct that may better reflect the overall burden of muscle dysfunction [33, 34]. Taken together, these findings support theoretical models proposing inflammaging as a systemic amplifier of musculoskeletal decline [6, 35, 36].

Although higher BMI was positively associated with SMFD‐scores, this relationship could reflect the contribution of greater muscle mass to total body weight, rather than a protective effect of adiposity per se [37]. Since BMI does not differentiate between lean and fat mass, it remains unclear whether higher BMI in our sample reflects preserved muscle or increased fat accumulation. Caloric intake remained positively associated with SMFD even in models adjusted for BMI, suggesting an independent role of nutritional intake in supporting muscle function.

Although caloric intake was positively associated with SMFD‐score, it should be interpreted with caution, as it does not fully capture the complexity of nutritional status in older adults; in particular, macronutrient composition may play a more critical role in muscle health. Moreover, caloric intake may also reflect an indirect marker of overall well‐being and health status rather than a purely nutritional determinant. Notably, while mild adiposity may provide energy reserves or mechanical loading that support muscle function [38], excess fat accumulation, particularly intramuscular fat infiltration (myosteatosis) has been associated with local inflammation and metabolic dysfunction within skeletal muscle [39, 40]. Future studies incorporating direct measures of body composition, such as dual‐energy X‐ray absorptiometry or imaging‐based assessments of muscle and fat compartments, are needed to clarify the respective roles of lean mass, adiposity and myosteatosis in SMFD trajectories.

Taken together, our findings illustrate a dynamic interaction in which aging and systemic inflammation contribute to the decline in SMFD‐scores, whereas adequate caloric intake appears to exert a positive effect. These opposing influences shape the trajectory of muscle dysfunction and impact physical performance and autonomy in later life. Incorporating inflammatory and nutritional biomarkers into SMFD‐based clinical models may not only improve early identification of individuals at increased risk for accelerated muscle aging but also support clinicians in developing tailored and potentially more effective interventions to preserve muscle function.

This study presents several strengths that enhance interpretability. First, the adoption of the SMFD‐score as an integrative measure of muscle health represents a key methodological innovation [4]. The SMFD‐score captures the multidimensional nature of muscle aging and allows for a more accurate assessment of functional decline compared to traditional sarcopenia‐based approaches.

Second, the longitudinal design offers a dynamic perspective of the aging process [41]. The use of repeated measures reduces temporal misalignment between exposure and outcomes and strengthens causal inference through repeated measures. Third, the InCHIANTI cohort, a well‐characterized, community‐dwelling older population representative of the Italian population by age and sex, enhances external validity and supports generalizability to similar aging populations. Despite these methodological limitations, this study provides solid longitudinal evidence that supports the role of chronic inflammation and nutritional adequacy as key determinants of muscle function trajectories in later life.

However, several limitations should be acknowledged. The observational design precludes definitive causal inferences and the possibility of residual confounding remains, particularly from variables not included in the statistical model (e.g., pharmacological treatments). Dietary intake was self‐reported using a food‐frequency questionnaire and is therefore subject to recall bias.

Nutritional intake could also represent an indirect marker of overall level of general health, as for example more robust subjects could be more prone to a more balanced and complete dietetic intake; on the contrary participants with poorer health status could eat in a less balanced way [42].

Inflammatory biomarkers were assessed every 2–3 years, which may have missed short‐term variability and underestimated the complexity of the inflammatory response in aging. Eventually, attrition and survivor bias may have influenced the longitudinal associations, as individuals with poorer health were more likely to drop out or die during follow‐up [43]. IL‐6 was measured using different assay methods across study waves, which may have introduced some degree of measurement variability; this factor may have partially affected longitudinal comparability.

Moreover, although the SMFD‐score integrates multiple domains of muscle function, we did not perform separate analyses on individual components (muscle mass, density, strength and power); given their intrinsic intercorrelation, such analyses may be affected by collinearity and the primary aim of our study was not to disentangle the contribution of each single component, but rather to evaluate whether the SMFD‐score, as a composite measure, could provide an overall informative assessment of the determinants and modulators of muscle aging.

Finally, the cut‐off values used to define SMFD‐score quintiles are specific to the InCHIANTI population and should be interpreted with caution, as their generalizability requires confirmation in independent cohorts.

## 5 Conclusions

This study shows that the SMFD‐score captures longitudinal declines in muscle function with aging and is associated with both systemic inflammation and nutritional status.

## Funding

This work was supported by Ministero della Salute (10.13039/501100003196; ICS 110.1/RS97.71), and the National Institute on Aging (10.13039/100000049; N01‐AG‐916413; N01‐AG‐5‐0002; N01‐AG‐821336; R01‐AG‐027012).

## Ethics Statement

The InCHIANTI study baseline was approved by the Ethical committee at INRCA, Ancona (protocol 14/ce, 28 February 2000) as the FU1 (protocol 45/01, 16 January 2001). InCHIANTI study FU2 and FU3 were approved by the Local Ethical Committee at Azienda Sanitaria Firenze (protocol n° 5/04, 12 May 2004). The study was conducted in accordance with the Declaration of Helsinki and approved by the Ethics Committee of INRCA in Ancona (Italy). Clinical Trial Registration: NCT01331512.

## Consent

Written informed consent was obtained from the subjects to participate in the Study at each time (baseline visit and follow‐ups).

## Conflicts of Interest

The authors declare no conflicts of interest.

## Supporting information


**Table S1:** Sex‐specific percentile cut‐offs (P20, P40, P60, and P80) used to define quintiles for each component of the SMFD‐score.
**Table S2:** Linear Mixed‐Effects Models examining SMFD‐score trajectories over time according to IL‐6 tertiles. Sensitivity analysis, the model did not consider subjects with a SMFD‐score equal to zero.
**Table S3:** Linear Mixed‐Effects Models examining SMFD‐score trajectories over time according to caloric intake tertiles. Sensitivity analysis, the model did not consider subjects with a SMFD‐score equal to zero.
**Table S4:** Linear Mixed‐Effects Models examining SMFD‐score trajectories over time according to IL‐6 tertiles, caloric intake tertiles and their interaction.
**Figure S1:** InCHIANTI study design and follow‐up procedures.

## Data Availability

The data that support the findings of this study are available from the corresponding author upon reasonable request.
