# Supplementary material for: Skeletal Muscle Function Deficit Longitudinal Associations With Inflammation and Caloric Intake in Elderly. The InCHIANTI Study
Source: J Cachexia Sarcopenia Muscle. 2026 Aug 5;17(4):e70353. doi: 10.1002/jcsm.70353 (PMC13439984; doi:10.1002/jcsm.70353)
Supplement: Supplementary file 1 [file JCSM-17-e70353-s001.docx]

Table S1. Sex-specific percentile cut-offs (P20, P40, P60, and P80) used to define quintiles for each component of the SMFD-score.

|  | Men | | | |  | Women | | | |
| --- | --- | --- | --- | --- | --- | --- | --- | --- | --- |
| **SMFD Component** | **P20** | **P40** | **P60** | **P80** |  | **P20** | **P40** | **P60** | **P80** |
| Calf muscle area (cm²), 66% of tibial length | 6115.2 | 6895.4 | 7509.8 | 8220.8 |  | 4873.6 | 5376.6 | 5886.7 | 6419.2 |
| Calf muscle density (mg/cm³),66% of tibial length | 68.1 | 70.4 | 72.1 | 74.2 |  | 67.7 | 70.1 | 71.9 | 73.9 |
| Isometric handgrip strength (Kg) | 20.0 | 38.0 | 45.0 | 52.5 |  | 17.5 | 22.0 | 25.5 | 31.0 |
| Peak lower limb extension power (W) | 100.0 | 146.0 | 187.1 | 240.7 |  | 46.8 | 67.0 | 90.0 | 128.9 |

P20 = cut-off between I and II quintile

P40 = cut-off between II and III quintile

P60 = cut-off between III and IV quintile

P80 = cut-off between IV and V quintile

Table S2: Linear Mixed-Effects Models examining SMFD-score trajectories over time according to IL-6 tertiles. Sensitivity analysis, the model did not consider subjects with a SMFD-score equal to zero.

|  |  | Model E |
| --- | --- | --- |
| Fixed effects |  |  |
| Intercept | γ _00_ | 2.43±0.81 ** |
| Time | γ _10_ | - |
| IL-6 tertiles | γ _20_ | 0.10±0.09 |
| IL-6 tertiles x Time | γ _30_ | -0.31±0.02 *** |
| Variance components |  |  |
| Within person | δ ^2^ _e_ | 5.91±0.24 *** |
| Intercept | δ ^2^_0_ | - |
| In rate of change | δ ^2^_1_ | - |
| In IL-6 tertiles | δ ^2^ _2_ | 0.04±0.04 |
| In IL-6 tertiles x Time | δ ^2^_3_ | 0.02±0.02 |
| AIC |  | 15870 |

The Model represents the fully adjusted model, including baseline age and sex, as well as time-varying covariates for multimorbidity, Body Mass Index (BMI), Cigarette smoking status, Alcohol-Drink habits, self-report physical activity level and caloric intake tertiles.

* *p* < .05; ** *p* < .01; *** *p* < .001.

Table S3: Linear Mixed-Effects Models examining SMFD-score trajectories over time according to caloric intake tertiles. Sensitivity analysis, the model did not consider subjects with a SMFD-score equal to zero.

|  |  | Model E |
| --- | --- | --- |
| Fixed effects |  |  |
| Intercept | γ _00_ | 2.13±0.80 ** |
| Time | γ _10_ | - |
| Caloric intake tertiles | γ _20_ | 1.21±0.15 *** |
| Caloric intake tertiles x Time | γ _30_ | 0.25±0.03 *** |
| Variance components |  |  |
| Within person | δ ^2^ _e_ | 5.95±0.25 *** |
| Intercept | δ ^2^_0_ | 7.85±0.97 * |
| In rate of change | δ ^2^_1_ | 0.19±0.11 * |
| AIC |  | 14893 |

The Model represents the fully adjusted model, including baseline age and sex, as well as time-varying covariates for multimorbidity, Body Mass Index (BMI), Cigarette smoking status, Alcohol-Drink habits, self-report physical activity level and IL-6 tertiles.

* *p* < .05; ** *p* < .01; *** *p* < .001.

Table S4: Linear Mixed-Effects Models examining SMFD-score trajectories over time according to IL-6 tertiles, caloric intake tertiles, and their interaction.

|  |  | Model Estimates | p-value |
| --- | --- | --- | --- |
| Fixed effects |  |  |  |
| Intercept | γ _00_ | 9.45±0.54 | <0.001 |
| IL-6 tertiles | γ _10_ | 0.30±0.26 | 0.25 |
| Caloric intake tertiles | γ _20_ | 1.49±0.29 | <0.001 |
| Caloric intake tertiles x Time | γ _30_ | -0.26±0.07 | <0.001 |
| IL-6 tertiles by Time | γ _40_ | -0.51±0.06 | <0.001 |
| IL-6 tertiles x Caloric intake tertiles | γ _50_ | -0.25±0.14 | 0.08 |
| IL-6 tertiles x Caloric intake tertiles x Time | γ _60_ | 0.14±0.04 | <0.001 |
| Variance components |  |  |  |
| Within person | δ ^2^ _e_ | 5.99±0.26 | <0.001 |
| Intercept | δ ^2^_0_ | 6.66±1.50 | <0.001 |
| In IL-6 tertiles | δ ^2^_2_ | 0.02±0.41 | 0.48 |
| In IL-6 tertiles x time | δ ^2^_3_ | 0.04±0.01 | 0.001 |

Figure S1. InCHIANTI study design and follow-up procedures.

Figure S1 Footnote: The InCHIANTI (Invecchiare in Chianti) study is a population-based longitudinal study conducted in Tuscany (Italy) to identify determinants of mobility decline in aging. Baseline data collection (1998–2000) included a comprehensive, standardized, multi-step assessment: 1) a home interview covering socio-demographic factors, lifestyle, disability, cognitive status, mood, and health behaviors; 2) a clinical and medical evaluation to ascertain diseases and pharmacological treatments; 3) an objective functional assessment focused on walking ability, balance, muscle strength, and range of motion; 4) an instrumental and laboratory session including anthropometry, pQCT, ECG, EMG, Doppler studies, and extensive blood and urine analyses, with biological samples stored for future research. Follow-up evaluations were systematically repeated using the same standardized protocol to ensure longitudinal comparability. Recruitment occurred between 1998 and 2000, with assessments repeated approximately every three years through 2018. This follow-up timetable enabled the study of trajectories of functional decline of the sub-system considered and the study of incident disability over time. To guarantee reproducibility and internal validity, all field researchers including interviewers, geriatricians, and physical therapists were specifically trained and supervised using standardized operating procedures consistent with the baseline protocol. Data quality was further ensured through rigorous data management procedures: information was entered using a double data-entry system, and any discrepancies were systematically checked against the original Case Report Forms.
